# On the Mode by Which Death Is Produced by Hanging

**Published:** 1814-12

**Authors:** 


*For the Medical and Physical Journal.*

### *On the Mode by which Death is produced by Hanging;* by MEDICUS.

THE circumstances attending the recent execution of the unfortunate Ashton, may be adverted to as containing an illustration of the mode by which Death is occasioned from Hanging. It has been generally supposed to arise from one of two causes, dislocation of the neck and suffocation; but the former supposition has, I believe, been generally abandoned. That it is not owing to suffocation alone, I presume, from the fact that death occasioned by strangling, however complete the act, is not so speedy as that produced at the ordinary execution *drops*.

The narrative of the case in the newspapers is this:—"The signal was given, and the platform fell: scarcely, however, had the other sufferers dropt, before, to the awe and astonishment of every beholder, Ashton rebounded from the rope, was instantly seen dancing near the ordinary, and clapped his hands and huzzaed. At length the executioner *pushed* him from the place, and he *met his fate in great agony*, and died in strong convulsions."

When the reporter speaks of his having rebounded *after having dropped*, he is evidently mistaken: it is more probable that at the instant of the platform falling, he was jumping towards the ordinary; and I can readily believe he was actually dancing by him while the other sufferers were suspended.

I believe death from hanging, as it is practised in London, to be produced solely by the violent shock inflicted on the spinal marrow at the juncture of the head and neck, as it is produced in the rabbit by the striking of the neck at that part. In the old system of drawing a cart from under the criminal, and in the case of Ashton, who was pushed off the scaffold, this effect would be in a great degree lessened, if not prevented, leaving the suffocation alone as the cause of death. It is to the perpendicular drop, and the immense shock shock occasioned by it, that the instantaneousness of the death is due.

The slight muscular movements seen in some who are executed by the drop, are no proofs of the continuance of feeling or vitality, but merely the remains of muscular irritability which exists even in parts separated from the body, and which may often be excited in dead bodies by the galvanic action. But these are very different, and not to be compared to the horrid struggles and convulsions which have been witnessed in executions by the old method.

